# The “Resus:Station”: The use of clinical simulations in a randomised crossover study to evaluate a novel resuscitation trolley^☆^

**DOI:** 10.1016/j.resuscitation.2012.06.026

**Published:** 2012-11

**Authors:** Susanna T. Walker, Stephen J. Brett, Anthony McKay, Rajesh Aggarwal, Charles Vincent

**Affiliations:** a Clinical Safety Research Unit, Department of Surgery & Cancer, Imperial College London, 10th Floor QEQM Building, St Mary's Hospital, Praed Street, London W2 1NY, UK; b Centre for Perioperative Medicine and Critical Care Research, Department of Anaesthesia and Intensive Care, Hammersmith Hospital, Imperial College Healthcare NHS Trust, Du Cane Road, London W12 0HS, UK; c Department of Resuscitation and Outreach, St Mary's Hospital, Imperial College Healthcare NHS Trust, Praed Street, London W2 1NY, UK; d Division of Surgery, Department of Surgery & Cancer, Imperial College London, 10th Floor QEQM Building, St Mary's Hospital, Praed Street, London W2 1NY, UK

**Keywords:** Resuscitation trolley, Clinical simulation, Patient safety, Equipment design, Team efficiency

## Abstract

**Background and aim:**

Inadequately designed equipment has been implicated in poor efficiency and critical incidents associated with resuscitation. A novel resuscitation trolley (Resus:Station) was designed and evaluated for impact on team efficiency, user opinion, and teamwork, compared with the standard trolley, in simulated cardiac arrest scenarios.

**Methods:**

Fifteen experienced cardiac arrest teams were recruited (45 participants). Teams performed recorded resuscitation simulations using new and conventional trolleys, with order of use randomised.

After each simulation, efficiency (“time to drugs”, un-locatable equipment, unnecessary drawer opening) and team performance (OSCAR) were assessed from the video recordings and participants were asked to complete questionnaires scoring various aspects of the trolley on a Likert scale.

**Results:**

Time to locate the drugs was significantly faster (*p* = 0.001) when using the Resus:Station (mean 5.19 s (SD 3.34)) than when using the standard trolley (26.81 s (SD16.05)).

There were no reports of missing equipment when using the Resus:Station. However, during four of the fifteen study sessions using the standard trolley participants were unable to find equipment, with an average of 6.75 unnecessary drawer openings per simulation.

User feedback results clearly indicated a highly significant preference for the newly designed Resus:Station for all aspects.

Teams performed equally well for all dimensions of team performance using both trolleys, despite it being their first exposure to the Resus:Station.

**Conclusion:**

We conclude that in this simulated environment, the new design of trolley is safe to use, and has the potential to improve efficiency at a resuscitation attempt.

## 1 Introduction

**Fig. 1:**
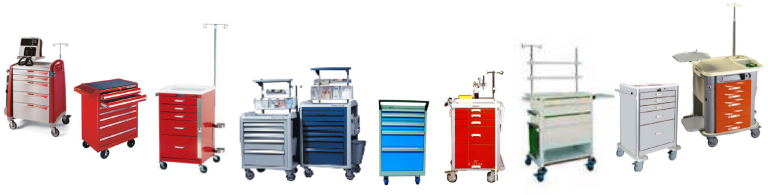
A selection of resuscitation trolleys commonly found in the UK.

When the members of a resuscitation team are summoned to treat a patient, they expect to be greeted with a fully stocked resuscitation trolley containing all the equipment required to treat the patient in those critical first few minutes. “Emergency Trays” for different procedures have been described since the early 1950s^1^ before the inception of CPR in the 1960s.^2^ Shortly after this, a “cart” was developed with equipment required for resuscitation, but whilst clinical care, and the equipment required, has progressed dramatically since this time, the design of the trolley itself remains largely the same as those used by mechanics as tool trolleys, and is not designed for purpose. Fig. 1 illustrates a selection of resuscitation trolleys commonly found in UK hospitals. All have the same basic design of a series of drawers in which to store all the equipment required.

In 2004, the Department of Health “Design for Patient Safety” report^3^ identified the need for a systems-level approach to design within the NHS, and in particular for collaboration between designers, researchers, healthcare practitioners and NHS agencies. They alluded to the fact that there is evidence to suggest that well-designed packaging, communications, and environments can reduce the incidence of errors within healthcare, and suggested that design should not be limited to obvious areas, but may be used to design out some common medical errors.

**Fig. 2:**
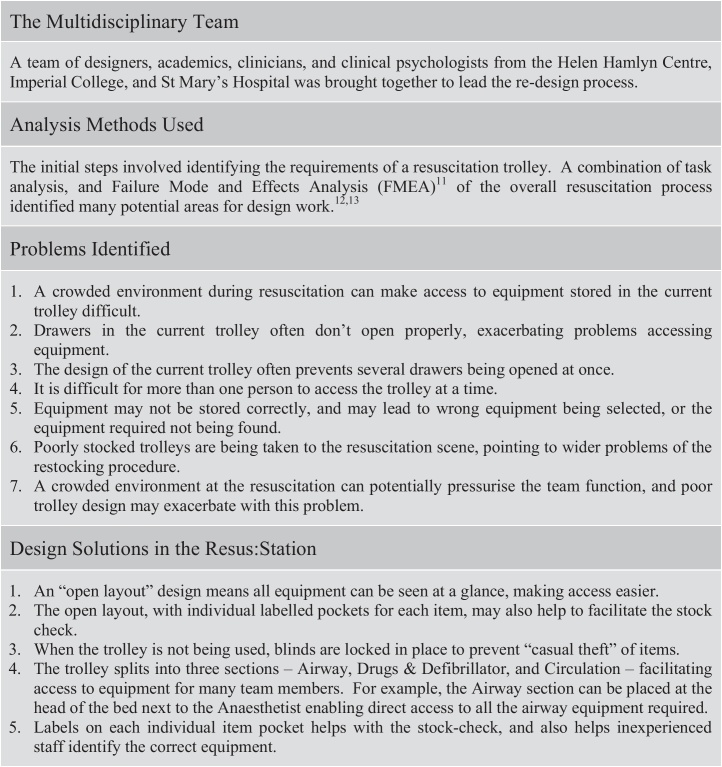
An brief explanation of the development process in designing the Resus:Station..

**Fig. 3:**
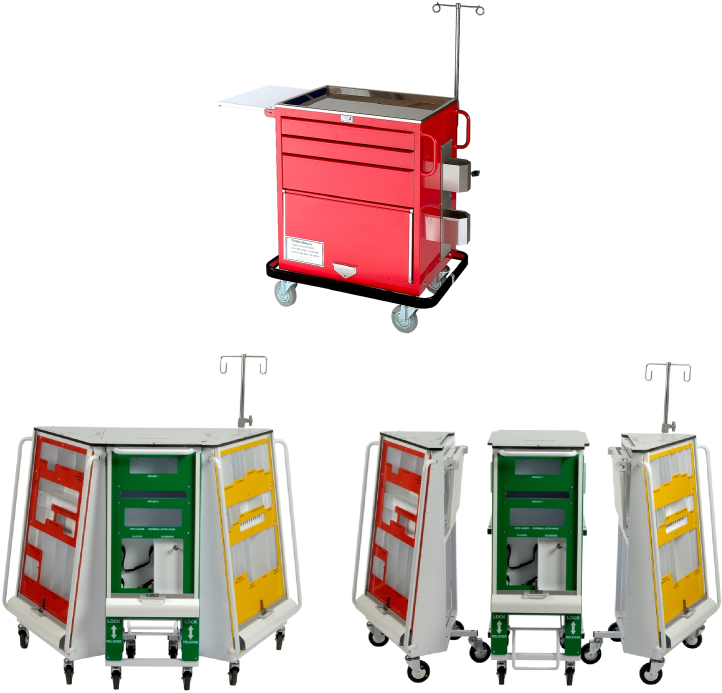
The standard resuscitation trolley from our hospital, and the newly designed Resus:Station prototype.

Shortly after this, in 2005, National Reporting and Learning System (NRLS) internal data, provided to us as part of this study, highlighted the fact that ill-stocked resuscitation trolleys had directly led to a number of preventable deaths. Furthermore, of eighty-six incidents involving resuscitation trolleys reported to the National Patient Safety Agency (NPSA) in 2005/6, thirteen were thought to have led to patient harm, and ten to have contributed directly to the deaths of patients. This issue was subsequently referred to in an open access publication from 2007.^4^ It is well recognised that the resuscitation process occurs in a high-stress, time pressured environment with a large ad hoc team, thus potentiating the risk of error.^5,6^ However, despite this, there are surprisingly few publications describing adverse events and critical incidents during resuscitation. Andersen et al.^7^ searched the Danish Patient Safety Database for critical incidents related to cardiac arrest management. Of incidents reviewed, 32% related to equipment issues, in similar areas to those reported by the NPSA. An earlier study by King et al.^8^ in 1994 found that 18% of cardiopulmonary resuscitation attempts were delayed by equipment failures. Most recently, Ornato et al.^9^ have reported results from the National Registry of Cardiopulmonary Resuscitation (NRCPR) database, and similarly report equipment issues as being one of the common causes of system errors during resuscitation. Therefore, whilst the NPSA recognised the importance of clinician training, they also felt there was a case to investigate the design of the resuscitation trolley itself, acknowledging the fact that ergonomic design can lead to a reduction in error.^10^ In light of this, the NPSA sponsored an initial redesign of the resuscitation trolley (Fig. 2). This led subsequently to the production of the “Resus:Station”, examined in this study (Fig. 3).

The aim of the study reported here was to assess the 2nd generation Resus:Station in a simulated environment to ensure it was safe to use prior to placement in the clinical environment. We specifically wanted to assess the following three aspects:

1. Differences in efficiency of the resuscitation attempt when using the Resus:Station, compared with the standard resuscitation trolley.
2. Attitudes, opinions, and beliefs of participants regarding the new Resus:Station, when compared with the standard resuscitation trolley.
3. Differences in team performance in terms of behaviours and non-technical skills when using the Resus:Station compared with the standard resuscitation trolley.

## 2 Methods

### 2.1 Study design

This was a randomised crossover simulation study to determine efficiency, experience, and team performance of cardiac arrest teams using both the standard and newly designed resuscitation trolley. Randomisation of teams, using a computerised random number generator (www.randomizer.org) was performed to determine the order in which trolleys were used for each team. The result of this was concealed from the researcher enrolling participants, and was revealed just prior to the start of each simulation session. Due to the nature of the study it was not possible to blind participants or researchers.

### 2.2 Participants

Small cardiac arrest teams consisting of an anaesthetist, a physician and a nurse were recruited for each simulation session. Eligible participants all had some experience of attending real resuscitation attempts, with eligible physicians having led such attempts. To help facilitate the resuscitation simulation, one member of the research team acted as an additional junior nurse. His only role in this study was to perform chest compressions when requested by the study participants, to enable the participants to perform other resuscitation tasks. At no point did he offer any other advice or assistance to the team, or interact with either trolley in any way. Fifteen teams, consisting of a total of 45 participants were recruited.

### 2.3 Procedure

Overall participants were asked to complete two short cardiac arrest simulations in our simulation centre, one using the newly designed Resus:Station and one using the standard resuscitation trolley used in our hospital, with which they were familiar.

Prior to each simulation, participants were given a standardised simulation scenario to read, and the simulation then began. All simulations were performed using a Laerdal SimMan 3G Patient Simulator (Laerdal Medical, Stavanger, Norway) offering the highest fidelity patient simulation currently available. Simulations were recorded using a SMOTS™ mobile recording system (Scotia UK plc, Edinburgh, UK), for the purposes of constructive feedback to participants, and to enable retrospective detailed analysis of the simulations.

Simulations were standardised as much as possible, with the research team following a protocol determining the presentation of clinical findings to participants, the result of DC cardioversion or defibrillation, and when to stop simulation. Despite this, each was inevitably marginally different depending on how the team responded to the clinical scenario. For example, some teams reacted to clinical signs faster than others. They were therefore often quicker at defibrillating, and overall these simulations tended to be shorter. The first simulation was always a scenario of a patient with a ruptured aortic aneurysm leading to a cardiac arrest, and the second simulation was a scenario of a patient with chest pain leading to a cardiac arrest. Each lasted approximately 5 min.

### 2.4 Measures

#### 2.4.1 Efficiency

To measure the efficiency of the resuscitation team, three clear markers were identified during the simulations. These were:

(a) *Time to drugs*—defined as the time in seconds from the moment one of the team members said “let's give some adrenaline”, or similar wording, to the time at which whoever was searching for the drugs had the packet in their hand.
(b) *Missing equipment*—defined as occasions during the simulations when a team member searched for an item of equipment, was unable to find it, and stated that the equipment was missing, despite perfectly stocked trolleys.
(c) *Unnecessary drawer openings*—this was only assessed when teams were using the standard trolley, as there are no drawers on the Resus:Station. An unnecessary drawer opening was defined as an occasion when a team member opened a drawer, looked inside, and closed the drawer without removing equipment, which clearly represents inefficient use of time.

Two expert clinical observers, an anaesthetist and a resuscitation officer, independently retrospectively made assessments of the team efficiency by watching the video recordings of each study session.

#### 2.4.2 Attitudes and views

After each simulation, irrespective of which trolley had been used, participants were asked to complete a questionnaire (Electronic Appendix A) giving their opinions to a series of statements about the trolley used. They were asked to rate how strongly they agreed or disagreed with the statement on a Likert scale of one to seven, with any additional comments in a free-text box. These comments were collated in the form of quotes. At the end of the session they were asked to complete a final questionnaire with a similar format asking them to state their preference of trolley, new or old.

#### 2.4.3 Team performance

We retrospectively rated the non-technical teamworking skills of every team during both simulations to determine whether a novel piece of equipment had any impact on these skills. Two researchers – an anaesthetist and a resuscitation officer – watched video recordings and rated behaviours using the “OSCAR” tool,^14^ (Electronic Appendix B) developed specifically for this purpose. This rates each individual team member's non-technical skill behaviours in six behaviour categories—Communication, Cooperation, Co-ordination, Leadership, Monitoring, and Decision Making. Assessors were kept blinded to each other's ratings during this process, and had been trained in the use of the tool prior to the beginning of the study.

### 2.5 Statistical analysis

All data analyses were carried out using SPSS v. 18.0 (SPSS Inc., Chicago, IL, USA). The tests used included calculation of mean (standard deviation) for parametric datasets, and median (IQR) for ordinal datasets such as questionnaire ratings; *t*-tests or non parametric equivalents were used as appropriate for group comparison. Intraclass correlation coefficients (ICCs) were used to assess inter-rater reliability of OSCAR scores. This test is recommended in the literature^15^ to measure the level of agreement between assessors using an assessment instrument, with ICC values of 0.70 or higher indicating adequate agreement.

## 3 Results

### 3.1 Efficiency

#### 3.1.1 Time to drugs

The time to administering drugs was significantly faster when using the Resus:Station compared with the standard trolley (mean 5.19 s (SD 3.34) versus mean 26.81 s (SD 16.05); mean difference 21.62 s (95% CI diff 11.48–31.76), *p* = 0.001). This time difference remains significant when analysing the sub-groups in terms of which trolley was used first; Resus:Station first (standard trolley mean 25.29 s (SD 18.49) versus Resus:Station mean 6.29 s (SD 3.45); mean difference 19.0 s (95% CI diff 1.09–36.91), *p* = 0.041) or standard trolley first (standard trolley mean 28.58 s (SD 14.19) versus Resus:Station mean 3.92 s (SD 3.0); mean difference 24.66 s (95% CI diff 9.64–39.70), *p* = 0.008).

#### 3.1.2 Missing equipment

There were no occasions when using the Resus:Station that equipment was reported missing. However, during four of the fifteen study sessions using the standard trolley team members were unable to find a piece of equipment on the trolley, specifically:

1. An intubating bougie.
2. Saline ampoules.
3. Amiodarone minijet.
4. The mask for a self-inflating bag.

On each occasion, the research team was able to find the equipment on the trolley for participants after the simulation had finished.

#### 3.1.3 Unnecessary drawer openings

During our short simulations, there was a median of 6.75 (IQR 2.13–9.38) wasted drawer openings when using the standard trolley. There are no drawers on the Resus:Station with which to make a direct comparison.

### 3.2 Attitudes and views

The overall user feedback results clearly indicate a strongly significant preference for the newly designed Resus:Station for all aspects (Table 1). This includes the enhancement of teamwork during a resuscitation attempt, the fact that equipment is easily accessible, and the fact that it is intuitive to use and could be used without instruction. The final questionnaire results report a strong preference for the ergonomically designed trolley (Table 2); for every aspect participants preferred the Resus:Station. This is particularly true for the final statement “To aid the restocking process” which not only has the lowest median score (median 1 (IQR 1–2)), but also has the smallest total range of responses (1–4), with no participants stating a preference for the standard trolley. The preference for the Resus:Station was apparent regardless of which trolley was used first. Table 3 lists a representative selection of quotes from participants about the Resus:Station, with positive quotes far greater in number than negative quotes.

### 3.3 Team performance

Having rated teams using the OSCAR tool, we then made a comparison of the median scores for each behaviour category when using both trolleys (Electronic Appendix C). Overall the teams performed equally well for all dimensions of team performance, on the new trolley and the old trolley, even though it was the first time they had ever seen the Resus:Station. This remained true when subanalysis looked at which trolley was used first, and also when looking at different team members—anaesthetists, physicians, and nurses. Scores from both assessors were compared to assess inter-rater reliability. Of the 18 scored behaviours (three team subgroups, and six behaviour categories) that the tool assesses, all achieved highly significant (*p* < 0.001) intraclass correlation coefficient (ICC) results, 12 of which were very high with results ≥0.70 (Electronic Appendix D).

## 4 Discussion

We have demonstrated improved efficiency when resuscitation teams are exposed to the new Resus:Station resuscitation trolley for the first time in a simulated environment. Study participants opinions were strongly in favour of the new trolley, and there were no detrimental effects on non-technical teamworking skills.

Historically, it has been unusual to conduct clinical trials of pieces of equipment. However, as has been highlighted in the “Design for Patient Safety” reports,^3^ there is an increasing appreciation of the links between design of equipment, human error, and patient safety issues. In 2001, the Joint Commission on Accreditation of Healthcare Organisations (JCAHO) in the US published a report^16^ looking at ways to improve patient safety standards. In this, Spath stated “*If healthcare is to improve patient safety, systems and processes must be designed to be more resistant to error occurrence and more accommodating of error consequence*”. Reason^17^ argues that processes that require perfect human performance are fatally flawed, and Grout^18^ goes on to coin the term “Mistake proofing” to change the physical design of a process to reduce human error. Having redesigned an item with these issues in mind, it remains important to demonstrate safety and benefit when compared with the previous standard. Whilst the NPSA has produced a guide to user testing in the development of medical devices^19^ we were unable to find examples of equipment testing in the literature. We have therefore developed a novel way to evaluate a piece of equipment in a simulated environment prior to introducing it to the ward, a process that is now being used for trials of other clinical equipment.

Whilst developing a protocol to assess efficiency of the resuscitation team, we looked in detail for consistent elements that were seen during resuscitations and were accurately measureable as surrogate markers of efficiency. A number of candidate measures, such as time to intubation, were assessed, but having examined the video recordings of simulations, they were found not to be robust, as they did not reliably have fixed points in time. There were a variety of reasons for this. For example, some related to the fact that many thought processes are not consciously verbalised, and therefore not possible to measure.

Our first reliable marker of efficiency was “Time to Drugs”. The use of adrenaline in resuscitation has recently been trialled by Jacobs et al.^20^ Although they were unable to demonstrate improved survival with the use of adrenaline, it remains in the guidelines and so the general issue of the time interval between decision and delivery of drugs seems a plausible marker of efficiency. We found it encouraging that medications were accessed significantly faster when using the Resus:Station in comparison to the standard trolley. Of note is the fact that this remained true even when analysing the subgroups in terms of trolley order of use. When performing two simulations consecutively there is likely to be a degree of learning of the process of the simulation. One anticipates the second simulation will run more smoothly as the participants understand what to expect. Therefore, it is significant that despite the possibility of a learning effect participants were able to find drugs significantly faster on the Resus:Station, even when this trolley was used first. This is especially encouraging since in their report initiating this project, the National Patient Safety Agency highlighted the fact that time is of the essence at a resuscitation attempt, and that time spent searching for equipment is an inefficient use of time and may potentially affect the patient's chances of survival. The potential importance of this finding was highlighted in Ornato et al.’s^9^ review of the NRCPR data and resuscitation system errors. In their results, they specifically demonstrate decreased survival when administration of adrenaline was delayed, and in their recommendations state that training should be targeted to avoid the types of errors that have the greatest impact on survival, including minimising delays in medication administration. We feel that this, and our data relating to equipment that was not found during simulations is particularly encouraging when one remembers the cumulative effect of staff searching for multiple pieces of equipment over the course of a prolonged resuscitation attempt. Difficulty finding equipment leads to members of staff being sent away to hunt elsewhere, leads to delays in delivery of care, may increase stress levels amongst team members, and may increase the rates of error and adverse events. This is on top of the emergency situation itself causing stress, which can affect performance, as discussed by Norris and Lockey.^21^ Importantly, Sandroni et al.,^22^ noted that even resuscitation courses lead to stress that causes indecision and delay.

Our final marker of efficiency identified the fact that when using the standard trolley, team members tend to open and close drawers successively without removing a piece of equipment whilst hunting for something. This happened an average of 6.75 times per simulation during our study. Given that the mean length of time of our simulation (5 min 51 s) is very short compared to the time spent at a real cardiac arrest, it is bewildering to imagine how many times drawers are opened unnecessarily at a clinical event. One participant is quoted as saying “There's one noise heard at arrests more than any other – the sound of those metal drawers slamming shut as people frantically search for what probably isn’t there”. This not only delays delivery of equipment, but also potentially increases the noise and stress levels at the resuscitation attempt; participants often commented that the simulation seemed calmer when using the Resus:Station rather than the standard trolley. Unnecessary noise has been shown by others to make errors more probable, and potentiate the risk of negative outcomes for patients.^23,24^

The results from the questionnaires capturing opinions of the new Resus:Station clearly demonstrated a strong preference for the new design. These results are even more powerful when it is remembered that the study session was the first time any of the participants had ever seen the new trolley. One might expect the fact that all participants were familiar with the standard trolley to bias results in its favour. Some participants commented on this fact, and stated that if they were to repeat the study having become familiar with the new trolley, they might score it even more favourably. We found it particularly interesting that study participants found the new trolley more intuitive to use than the trolley with which they are already familiar. This was not an outcome that had been anticipated, but confirms the success of the ergonomic design.^3,10^ It was also reassuring that the possible effect of learning from performing two simulations in quick succession did not have a significant impact on the questionnaire results.

We did not demonstrate any significant difference in the non-technical teamworking skills of teams when using the two trolleys. This was despite it being the first time teams had ever seen the Resus:Station. Our aim was specifically to demonstrate no detrimental effect to provide reassurance that it is safe to use in a clinical environment. Interestingly, when participants were asked whether they thought the new trolley had any effect on team-working, some commented that there was less communication when using the ergonomically designed trolley, and that team members tended to get on with their own tasks without communicating to others. This was interpreted by some as being detrimental to teamwork during the resuscitation. However, not all communication contributes to optimal team function^14^—especially if it concerns the pursuit of elusive items of equipment. Flin et al.^25^ refer to what the US naval air-service term “comm-brevity”, emphasising that during periods of high workload, only the most relevant information should be given to preserve cognitive resources of the sender and the receiver. This is something we plan to analyse in greater detail in a separate study, as in the long-term it is likely that the new trolley may actually improve teamwork.

Finally, in subanalysis of the questionnaire data, the majority of the statements for which there was less of a significant difference in responses between the two trolleys related to aesthetic design of the trolley, and whether participants felt the trolley had flaws in its design. Participants had fewer concerns about what the trolley actually looked like than its functionality. The Resus:Station trolley used for the purposes of the study was a prototype. Lessons learnt from the simulations have guided subsequent design refinement and development of the next prototype prior to manufacture of the final product.

### 4.1 Limitations

The main limitation of this study is that the small cardiac arrest team, and short simulation scenario limited the possibilities for efficiency assessment. We would ideally repeat this at a real arrest with a full resuscitation team. However, this small study has enabled us to have some insight into the potential impact of an ergonomically designed resuscitation trolley.

## 5 Conclusion

In conclusion, we have described a thorough process of verification of the Resus:Station in a simulated environment when comparing it to a standard resuscitation trolley. We have demonstrated strongly positive results in terms of team efficiency and user opinion, and no detrimental effect on non-technical team working skills. We can therefore conclude that in this environment, the new design of trolley is safe to use, and has the potential to improve efficiency at a resuscitation attempt. However, the potential impact may be subtle, and would require a large, multicentre clinical study to demonstrate an effect on patient outcomes.

## Conflict of interest statement

S. Brett is a co-author on the worksheet “Quality of life after resuscitation” in the 2010 guideline revision. He has a research grant from Carefusion, and consults for Pfizer and Baxter Healthcare.

No other conflict of interest is declared.

## Ethics statement

Ethics permission for this study was awarded by the North West London Research Ethics Committee on 4th November 2008. Study title: A clinical study of a novel resuscitation trolley, REC reference: 08/H0722/91.

## Figures and Tables

**Table 1:** Participant responses to the instruction “Please think about the resuscitation trolley that you used in the simulation you have just completed. Now read the following statements about this trolley and rate your answers according to how strongly you agree or disagree with each statement”. 1 = strongly disagree; 4 = neither; 7 = strongly agree.

Question	Overall data		
	Trolley used – median (IQR)		Significance of difference
	Standard	Resus:Station	
Overall this is an excellent trolley with no problems	3.0 (1.5–4)	5.0 (4–6)	<0.001
It is very easy to find equipment on this trolley	3.0 (2–5)	5.0 (5–6)	<0.001
I think it would be easy to check and stock this trolley	3.0 (2–5)	6.0 (5.25–7)	<0.001
The design of this trolley makes my role in resuscitation easier	3.0 (2–4)	5.0 (4.5–6)	<0.001
All the equipment required for intubation is easily accessible	4.0 (2–5)	6.0 (5–6)	<0.001
The aesthetic design of this trolley is appealing	4.0 (3–4)	5.0 (4–6)	<0.001
This trolley enhances teamwork in resuscitation	3.0 (2–4)	4.5 (4–6)	<0.001
The design of this trolley is intuitive and its use does not need explaining	3.0 (2–4)	5.0 (5–6)	<0.001
All the drugs and fluids required for resuscitation are easy to find	3.0 (2–4)	5.5 (5–6)	<0.001
There is sufficient workspace on top to lay equipment out if required	3.0 (1–3)	6.0 (5–6)	<0.001
I would be able to use this trolley without instruction	3.0 (2–5)	6.0 (5–6)	<0.001
This trolley significantly contributes to a successful resuscitation	3.0 (2–4)	5.0 (4–6)	<0.001
This trolley could be better designed	6.0 (5–7)	5.0 (4–6)	<0.001
This trolley would benefit from a review of its design	6.0 (5–7)	5.0 (3–5)	<0.001
There are flaws I can identify in this trolley	6.0 (5–7)	5.0 (3.25–5)	<0.001

**Table 2:** Participant responses to the statement “When considering the following factors, please indicate on the scale which trolley you prefer”. 1 = definitely Resus:Station; 4 = no preference; 7 = definitely standard trolley.

Question	Score – median (IQR)
Overall use	2 (1–3)
Ease of finding equipment	2 (1–3.5)
Aesthetic design	2 (1–3.5)
To facilitate teamwork	3 (2–4)
To facilitate running an efficient resuscitation	2 (1.5–3)
To aid the restocking process	1 (1–2)

**Table 3:** A representative selection of quotes from study participants about the Resus:Station.

Positive Quotes about the Resus:Station
“I like the way you can see everything immediately.”

“I’m familiar with the old trolley and therefore found it easier to use. However, I like the colour coded sections of the Resus:Station, and thought the labelling of items could facilitate easier retrieval for people less familiar with resuscitation equipment.”

“Normally you’re stressed finding things; you’re throwing things in the air and asking for things. It's not efficient communication asking for things. Now that time can be used to communicate efficiently with each other.”

“I love it.”

“It's miles better than the current trolley.”

“I like the fact that we (*Anaesthetists*) can take the airway stuff, and not be “faffing” with everyone else around the trolley.”

Negative Quotes about the Resus:Station
“The trolley is huge – storage may be an issue – could it be made smaller in any way?”

“It's a great idea, and visually it's perfect, but it's too big.”

“Because the trolley was taken to the left of the bed (*as you look at the bed*), the airway section was nearest the feet, and so then there was a jumble trying to get the trolley to me (*the anaesthetist*).”

“There's a lot of wasted space in the trolley.”
