# Supplementary material for: The “Resus:Station”: The use of clinical simulations in a randomised crossover study to evaluate a novel resuscitation trolley
Source: Resuscitation. 2012 Nov;83(11):1374–80. doi: 10.1016/j.resuscitation.2012.06.026 (PMC3482665; doi:10.1016/j.resuscitation.2012.06.026)
Supplement: Supplementary file 1 [file mmc1.docx]

**Supplementary Online Material**

**Electronic Appendix A – Post-simulation questionnaire given to study participants**

Date: Participant code: Simulation number:

**Trolley Questions:**

Please think about the resuscitation trolley that you used in the simulation you have just completed.

Now read the following statements about this trolley and rate your answers according to how strongly you agree or disagree with each statement.

|  | Strongly Disagree | | Neither | | | | | Strongly Agree | |
| --- | --- | --- | --- | --- | --- | --- | --- | --- | --- |
| Overall this is an excellent trolley with no problems | 1 | 2 | | 3 | 4 | 5 | 6 | | 7 |
| It is very easy to find equipment on this trolley | 1 | 2 | | 3 | 4 | 5 | 6 | | 7 |
| I think it would be easy to check and stock this trolley | 1 | 2 | | 3 | 4 | 5 | 6 | | 7 |
| The design of this trolley makes my role in resuscitation easier | 1 | 2 | | 3 | 4 | 5 | 6 | | 7 |
| All the equipment required for intubation is easily accessible | 1 | 2 | | 3 | 4 | 5 | 6 | | 7 |
| The aesthetic design of this trolley is appealing | 1 | 2 | | 3 | 4 | 5 | 6 | | 7 |
| This trolley enhances teamwork in resuscitation | 1 | 2 | | 3 | 4 | 5 | 6 | | 7 |
| The design of this trolley is intuitive and its use does not need explaining | 1 | 2 | | 3 | 4 | 5 | 6 | | 7 |
| All the drugs and fluids required for resuscitation are easy to find | 1 | 2 | | 3 | 4 | 5 | 6 | | 7 |
| There is sufficient workspace on top to lay equipment out if required | 1 | 2 | | 3 | 4 | 5 | 6 | | 7 |
| I would be able to use this trolley without instruction | 1 | 2 | | 3 | 4 | 5 | 6 | | 7 |
| This trolley significantly contributes to a successful resuscitation | 1 | 2 | | 3 | 4 | 5 | 6 | | 7 |
| This trolley could be better designed | 1 | 2 | | 3 | 4 | 5 | 6 | | 7 |
| This trolley would benefit from a review of its design | 1 | 2 | | 3 | 4 | 5 | 6 | | 7 |
| There are flaws I can identify in this trolley | 1 | 2 | | 3 | 4 | 5 | 6 | | 7 |

**Electronic Appendix B – The OSCAR tool**

**Observational Skill-based Clinical Assessment Tool for Resuscitation (OSCAR)**

**Date: Assessor: Candidate:**

0 = Team Severely Compromised 1 = Team Compromised

2 = Slight detriment to team 3 = Team neither enhanced or hindered

4 = Moderate enhancement to team 5 = High level of enhancement to team

6 = Highly effective in enhancing teamwork

**COMMUNICATION**

| **Anaesthetic Group (A)** | **Individual Behaviour Ratings** | | | | | | | **Global Behaviour Score (0-6)** |
| --- | --- | --- | --- | --- | --- | --- | --- | --- |
| Informs team whether patient is making respiratory effort | 0 | 1 | 2 | 3 | 4 | 5 | 6 |  |
| Informs team of any other relevant clinical signs eg dilated pupil, obvious injuries, signs of aspiration | 0 | 1 | 2 | 3 | 4 | 5 | 6 |  |
| Communication to team that they plan to intubate the patient if required | 0 | 1 | 2 | 3 | 4 | 5 | 6 |  |
| Requests patient history on arrival and communicates details to team, if required | 0 | 1 | 2 | 3 | 4 | 5 | 6 |  |
| **Physician Group (P)** | | | | | | | | |
| Reviews patient history and notes and communicates relevant details clearly to the team | 0 | 1 | 2 | 3 | 4 | 5 | 6 |  |
| Clear instructions communicated to the team regarding the arrest protocol | 0 | 1 | 2 | 3 | 4 | 5 | 6 |  |
| Encourages communication from sub-teams, and encourages team members to give opinions | 0 | 1 | 2 | 3 | 4 | 5 | 6 |  |
| **Nurse Group (N)** | | | | | | | | |
| Provides clear information about arrest events on arrival of arrest team | 0 | 1 | 2 | 3 | 4 | 5 | 6 |  |
| Senior nurse provides clear, audible requests to junior nurse when requesting equipment eg additional iv bags | 0 | 1 | 2 | 3 | 4 | 5 | 6 |  |
| Instructs other nurses on ward clearly how to assist with arrest or other ward duties as appropriate | 0 | 1 | 2 | 3 | 4 | 5 | 6 |  |

**CO-OPERATION**

| **Anaesthetic Group (A)** | **Individual Behaviour Ratings** | | | | | | | **Global Behaviour Score (0-6)** |
| --- | --- | --- | --- | --- | --- | --- | --- | --- |
| A-group provides information on request from P-group (eg about the airway) | 0 | 1 | 2 | 3 | 4 | 5 | 6 |  |
| A-group assists P-group in decision making in difficult scenarios | 0 | 1 | 2 | 3 | 4 | 5 | 6 |  |
| **Physician Group (P)** | | | | | | | | |
| Responds to questions from other team members about decisions made regarding the arrest | 0 | 1 | 2 | 3 | 4 | 5 | 6 |  |
| Supports less experienced members of P-group, and compensates for their lack of experience | 0 | 1 | 2 | 3 | 4 | 5 | 6 |  |
| **Nurse Group (N)** | | | | | | | | |
| Provide support and assistance to A-group and P-group when needed eg finding airway adjuncts | 0 | 1 | 2 | 3 | 4 | 5 | 6 |  |
| Help P-group locate items not routinely stocked on trolley, or missing from the trolley | 0 | 1 | 2 | 3 | 4 | 5 | 6 |  |
| Assist P-group with extra tasks eg sending bloods, contacting family, contacting labs etc | 0 | 1 | 2 | 3 | 4 | 5 | 6 |  |

**CO-ORDINATION**

| **Anaesthetic Group (A)** | **Individual Behaviour Ratings** | | | | | | | **Global Behaviour Score (0-6)** |
| --- | --- | --- | --- | --- | --- | --- | --- | --- |
| Information provided about changes in patient condition as they occur | 0 | 1 | 2 | 3 | 4 | 5 | 6 |  |
| A-group co-ordinate team to move patient eg floor to bed, up bed | 0 | 1 | 2 | 3 | 4 | 5 | 6 |  |
| **Physician Group (P)** | | | | | | | | |
| Notifies N and A groups of anticipated further requirements for patient resuscitation | 0 | 1 | 2 | 3 | 4 | 5 | 6 |  |
| Within P group, co-ordinates tasks such as taking of bloods, sending samples, sending ABG etc | 0 | 1 | 2 | 3 | 4 | 5 | 6 |  |
| **Nurse Group (N)** | | | | | | | | |
| Prepare Resus Trolley for use by team by bringing to bedside, turning monitor on etc | 0 | 1 | 2 | 3 | 4 | 5 | 6 |  |
| Prepare further drugs in readiness for their next required use eg prepare next adrenaline minijet | 0 | 1 | 2 | 3 | 4 | 5 | 6 |  |
| A Senior Nurse (Sister) is always present to provide backup to Staff Nurse | 0 | 1 | 2 | 3 | 4 | 5 | 6 |  |

**LEADERSHIP**

| **Anaesthetic Group (A)** | **Individual Behaviour Ratings** | | | | | | | **Global Behaviour Score (0-6)** |
| --- | --- | --- | --- | --- | --- | --- | --- | --- |
| Advises team on best management, and contingency plans for patient, and takes lead if required | 0 | 1 | 2 | 3 | 4 | 5 | 6 |  |
| Anaesthetist assertively takes a lead in Airway control and Ventilation on arrival at arrest | 0 | 1 | 2 | 3 | 4 | 5 | 6 |  |
| Lead Anaesthetist supervises and supports staff lacking familiarity with tasks or equipment | 0 | 1 | 2 | 3 | 4 | 5 | 6 |  |
| **Physician Group (P)** | | | | | | | | |
| Takes a lead and clearly instructs assistants with requirements for arrest and/or defers leadership as required if appropriate | 0 | 1 | 2 | 3 | 4 | 5 | 6 |  |
| Supervision given to staff lacking experience or familiarity with tasks or equipment | 0 | 1 | 2 | 3 | 4 | 5 | 6 |  |
| Instructs N-group of additional requirements eg recent blood results from computer, to call the family | 0 | 1 | 2 | 3 | 4 | 5 | 6 |  |
| **Nurse Group (N)** | | | | | | | | |
| Takes a lead with initial Basic Life Support attempts until Arrest Team arrive | 0 | 1 | 2 | 3 | 4 | 5 | 6 |  |
| Supervision and support given to junior or inexperienced members of N-team | 0 | 1 | 2 | 3 | 4 | 5 | 6 |  |

**MONITORING**

| **Anaesthetic Group (A)** | **Individual Behaviour Ratings** | | | | | | | **Global Behaviour Score (0-6)** |
| --- | --- | --- | --- | --- | --- | --- | --- | --- |
| Maintains monitoring of patient condition, signs of respiration, other clinical signs | 0 | 1 | 2 | 3 | 4 | 5 | 6 |  |
| Checks ventilation is adequate with regular blood gas analysis and amends ventilation accordingly | 0 | 1 | 2 | 3 | 4 | 5 | 6 |  |
| Confirms drug identity by checking syringe labeling prior to drug administration | 0 | 1 | 2 | 3 | 4 | 5 | 6 |  |
| **Physician Group (P)** | | | | | | | | |
| Maintains awareness of activities of other teams eg anaesthetist intubating | 0 | 1 | 2 | 3 | 4 | 5 | 6 |  |
| Monitors progress of resuscitation protocol with careful checking of time, and constant reassessment of limb of protocol and “extra considerations” | 0 | 1 | 2 | 3 | 4 | 5 | 6 |  |
| Checks team condition eg monitors for fatigue in team members from CPR and suggests team members change roles, take turns etc | 0 | 1 | 2 | 3 | 4 | 5 | 6 |  |
| **Nurse Group (N)** | | | | | | | | |
| Monitors patient dignity and considers well-being of other patients nearby | 0 | 1 | 2 | 3 | 4 | 5 | 6 |  |
| Maintains awareness of the needs of P and A groups | 0 | 1 | 2 | 3 | 4 | 5 | 6 |  |

**DECISION MAKING**

| **Anaesthetic Group (A)** | **Individual Behaviour Ratings** | | | | | | | **Global Behaviour Score (0-6)** |
| --- | --- | --- | --- | --- | --- | --- | --- | --- |
| Prompt identification of the problem | 0 | 1 | 2 | 3 | 4 | 5 | 6 |  |
| Rapidly and clearly outlines a strategy or plan, and asks for equipment | 0 | 1 | 2 | 3 | 4 | 5 | 6 |  |
| Anticipates potential problems and prepares accordingly – eg asks for further blood to be cross-matched | 0 | 1 | 2 | 3 | 4 | 5 | 6 |  |
| **Physician Group (P)** | | | | | | | | |
| Rapidly decides an appropriate course of action for continued resuscitation | 0 | 1 | 2 | 3 | 4 | 5 | 6 |  |
| Uses the team as a whole to help develop options – asks for opinions and processes them decisively | 0 | 1 | 2 | 3 | 4 | 5 | 6 |  |
| **Nurse Group (N)** | | | | | | | | |
| Prompt decision making during initial resuscitation attempts | 0 | 1 | 2 | 3 | 4 | 5 | 6 |  |
| Anticipates potential problems A and P teams may encounter eg pulls bed out from wall, clears area etc | 0 | 1 | 2 | 3 | 4 | 5 | 6 |  |
| Appropriate decision making regarding timing of initial decision to put out a cardiac arrest call | 0 | 1 | 2 | 3 | 4 | 5 | 6 |  |

**Electronic Appendix C – Overall results for the whole team using OSCAR to rate behaviours, with Wilcoxon assessment of the significance of difference when using the different trolleys. (* = result that just achieves significance)**

| **Behaviour** | | **Overall Scores (median IQR))** | | |
| --- | --- | --- | --- | --- |
|  |  | **Standard** | **Resus:Station** | **Wilcoxon** |
| Communication | 1 | 4 (3.5-4.5) | 4 (3.5-5) | 0.205 |
|  | 2 | 4 (3.5-5) | 4.5 (3.5-5) | 0.182 |
|  | 3 | 4 (3.25-4.5) | 4.5 (3.25-5) | 0.062 |
|  | 4 | 4 (3.5-5) | 4 (4-5) | 0.112 |
| Co-operation | 1 | 4.5 (4-5) | 4 (3-6) | 0.217 |
|  | 2 | 4 (3.5-4.75) | 4.5 (4-5) | 0.084 |
|  | 3 | 4 (3.5-5) | 4.5 (3.5-5) | 0.874 |
| Co-ordination | 1 | 4.5 (3.5-5) | 4 (3.5-5) | 0.256 |
|  | 2 | 3.5 (3-4.5) | 4.5 (3.75-5) | 0.049* |
|  | 3 | 3.5 (3-4) | 4 (3-4.75) | 0.586 |
| Leadership | 1 | 4 (3.25-5) | 4 (3.5-5) | 0.636 |
|  | 2 | 3.5 (3.5-5) | 4.5 (3.5-5) | 0.433 |
|  | 3 | 3.5 (3-4.625) | 4 (3.5-4.5) | 0.246 |
| Monitoring | 1 | 4 (3.5-4.5) | 4 (3-5) | 1.0 |
|  | 2 | 4 (3.5-4.5) | 4 (3.5-5) | 0.367 |
|  | 3 | 3 (3-4) | 3 (3-4) | 1.0 |
| Decision Making | 1 | 4 (3.5-5) | 4.75 (3.5-5) | 0.751 |
|  | 2 | 4 (3.5-5) | 4 (3.5-5) | 1.0 |
|  | 3 | 3 (3-4) | 3.5 (3-4) | 0.107 |

**Electronic Appendix D. OSCAR intraclass correlation coefficients between the two assessors across behaviours and subgroups for all simulations (n=30) (**P<0.001)**

|  | **Behaviour / Skill** | | | | | |
| --- | --- | --- | --- | --- | --- | --- |
| **Team Subgroup** | Communication | Co-operation | Co-ordination | Leadership | Monitoring | Decision Making |
| Anaesthetists | 0.71** | 0.57** | 0.71** | 0.62** | 0.70** | 0.57** |
| Physicians | 0.93** | 0.90** | 0.91** | 0.91** | 0.87** | 0.88** |
| Nurses | 0.76** | 0.64** | 0.55** | 0.81** | 0.69** | 0.81** |
